# Recent Graduates

**Published:** 1891-03

**Authors:** 


### Editorial,

### RECENT GRADUATES.

Within the next few weeks hundreds of young M. Ds. will be graduated from the numerous medical colleges in this country—many of them after having attended only two courses of lectures of five or six months each, with little or no preliminary instruction. Some of them without ever having stopped to consider the grave responsibilities which they are so lightly assuming. Of these latter many will soon drop by the wayside. They find themselves in an uncongenial atmosphere, where they cannot grow and develop, so they seek other and more pleasing occupations.

But there are others, and they are greatly in the majority, who realize the high calling of the profession, and are willing to work for the good of humanity and the development of science. They do not expect to amass wealth, but what is far higher and better, they expect to decrease the sum total of suffering and sorrow. They are prepared for lives of self-denial and devotion to duty, though beset by temptations on every hand—temptations far more powerful and insidious than any other profession is subjected to. The laity know little of medicines or of their effects on the animal economy, and the temptation to cover up inexcusable mistakes is great. The physician is brought into the most intimate relations with his patients, and it should be ever uppermost in his mind that the obligation to protect their morals is no less solemn and binding than is the obligation to minister to their physical infirmities.

The physician who becomes too much engrossed with the mere money-making side of his profession, who devotes a large proportion of his best thought and energies to making judicious investments, will soon find that his patients will conclude that he cares more for money than he does for medicine. The men who realize the highest and best that the profession offers them must ever keep paramount the good of humanity and the development of the healing art. They may not accumulate money; they may not become famous; their lives may often seem to them to be failures, but the world will be better for their having lived in it. They who accomplish less than this are failures, no matter how much of apparent success may have been attained.

